# The mitochondrial unfolded protein response (UPR^mt^) protects against osteoarthritis

**DOI:** 10.1038/s12276-022-00885-y

**Published:** 2022-11-15

**Authors:** Zhibin Zhou, Jiajia Lu, Mei Yang, Jiao Cai, Qiang Fu, Jun Ma, Lei Zhu

**Affiliations:** 1 Department of Orthopedics, General Hospital of Northern Theater Command, Shenyang, Liaoning China; 2 Department of Orthopedics, Second Affiliated Hospital of Navy Medical University, Shanghai, China; 3 Department of Anesthesia, Second Affiliated Hospital of Naval Medical University, Shanghai, China; 4 Department of Medical Administration, Second Affiliated Hospital of Navy Medical University, Shanghai, China; 5 Naval Medical Center of PLA, Naval Medical University, Shanghai, China

**Keywords:** Apoptosis, Bone

## Abstract

The mitochondrial unfolded protein response (UPR^mt^) is a mitochondrial-to-nuclear signaling pathway that is activated to maintain mitochondrial function when there is an accumulation of misfolded proteins within mitochondria. Mitochondrial function is essential for chondrocyte homeostasis, and mitochondrial dysfunction is a characteristic of osteoarthritis (OA). However, the role of the UPR^mt^ in OA remains unclear. In the present study, the level of the UPR^mt^ was examined in primary mouse chondrocytes subjected to different stresses and in the articular cartilage of OA model mice and OA patients. The relationship between UPR^mt^ activation and OA progression was studied. The UPR^mt^ was induced in primary mouse chondrocytes subjected to diverse stresses and in the cartilage of OA mice. Enhancement of the UPR^mt^ with nicotinamide riboside (NR) significantly improved mitochondrial function, reduced chondrocyte death, attenuated OA pain, and ameliorated OA progression, and the protective effects decreased significantly in chondrocyte-specific Atf5 knockout (*ATF5*^*f/f*^*Col2a1-CreER*^*T2*^) mice. UPR^mt^ induction was also identified in the articular cartilage of OA patients and was associated with reduced chondrocyte death, less severe hip pain, and lower levels of inflammation in synovial fluid. These findings identify the induction of the UPR^mt^ in primary mouse chondrocytes exposed to pathological stresses and in the articular cartilage of OA model mice and OA patients. Enhancement of the UPR^mt^ ameliorates OA progression, suggesting that the UPR^mt^ exerts a protective effect against OA and may be a potential diagnostic and therapeutic strategy for OA.

## Osteoarthritis: stress response in mitochondria protects against cartilage destruction

A cellular defense mechanism that helps preserve the function of mitochondria in the face of stressful stimuli also exerts a protective effect against cartilage degeneration, a key feature of OA. Using mouse cartilage cells, a team led by Jun Ma and Lei Zhu from the Naval Medical University in Shanghai, China, showed that the so-called mitochondrial unfolded protein response is induced by various molecular stimuli. Supplementing the diet with a form of vitamin B3 further enhanced this response, helping to promote mitochondrial function and ease disease-linked symptoms in a mouse OA model. A similar therapeutic strategy could be beneficial in patients, as the researchers showed that people with OA who have more active stress-response pathways in their mitochondria tend to have less cartilage degeneration, less severe hip pain, and lower levels of joint inflammation.

## Introduction

Osteoarthritis (OA) is the most common age-related joint disease and has a high morbidity rate in the elderly population^1^. However, there is a lack of effective therapies for OA due to our limited understanding of the disease mechanism^2^. To learn more about the pathophysiology of OA, the unique biological characteristics of cartilage, which is composed of chondrocytes and the surrounding extracellular matrix (ECM), should be studied in depth. Cartilage is usually regarded as hypoxic, and oxygen delivered to cartilage contains less oxygen than that delivered to other tissues^3^. It is known that mitochondria are extremely sensitive to hypoxic environments. Consequently, mitochondria play crucial roles in chondrocyte function through ATP generation; the metabolism of nucleotides, amino acids, and lipids; and the regulation of apoptosis and autophagy^4–6^. Mitochondrial dysfunction is a key feature of chondrocyte homeostasis disturbances and contributes to enhanced inflammation, increased cell death, decreased anabolic activity, and increased catabolic activity in OA. The vast majority of mitochondrial proteins are encoded in the nucleus, translated by cytosolic ribosomes, and imported into mitochondria. Both imported and mitochondrially synthesized proteins are folded correctly under the regulation of the mitochondrial protein quality control system to maintain mitochondrial proteostasis. Under conditions of environmental stress in OA, the mitochondrial protein folding environment is challenged, resulting in the accumulation of misfolded proteins and thus reducing mitochondrial respiration and inducing excessive production of reactive oxygen species^7^. Oxidative stress in turn increases the inflammatory response and results in progressive mitochondrial damage and dysfunction^8^.

Numerous mechanisms and signaling pathways have evolved to monitor proper mitochondrial function under stress. One of these mechanisms is the mitochondrial unfolded protein response (UPR^mt^)^9^, a mitochondria-to-nuclear response that is activated when mitochondrial proteostasis is disrupted and maintains normal mitochondrial function by increasing the transcription of chaperones and proteases to control protein folding, assembly, and degradation. These factors include heat shock 10 kDa protein (Hsp10), heat shock 60 kDa protein (Hsp60), ATP-dependent Clp protease proteolytic subunit (ClpP), Lon protease homolog mitochondrial (LonP1) and mitochondrial presequence translocase-associated motor complex protein (mtDNAj). The UPR^mt^ was first discovered in mammalian cells and comprehensively characterized in *C. elegans*^10^. Mitochondrial protein stress regulates the mammalian transcription factor ATF5 to induce a transcriptional response and rescue the UPR^mt ^^11^. The UPR^mt^ has been shown to play cytoprotective roles in the context of infection^12^ and cardiac stress^13^. However, the potential role of the UPR^mt^ in OA progression is unclear.

Here, we studied the relationship between UPR^mt^ activation and OA progression. We identified the induction of the UPR^mt^ in primary mouse chondrocytes subjected to different pathological stresses and in the articular cartilage of OA model mice and OA patients. We showed that enhancing the UPR^mt^ with nicotinamide riboside improved mitochondrial function in primary mouse chondrocytes and ameliorated OA progression in vitro and in vivo. In patients with OA, elevated levels of UPR^mt^ activation were associated with less severe clinical symptoms and pathological features. Taken together, our findings reveal an important protective role for the UPR^mt^ in the progression of OA and provide a potential diagnostic and therapeutic strategy for OA.

## Materials and methods

### Study approval

This study conformed to the principles of the Declaration of Helsinki, was approved by the Ethics Committee of Changzheng Hospital, and was performed in accordance with the committee’s guidelines.

### Patients and specimens

Twenty patients with hip OA and 20 with femoral neck fractures were recruited at Changzheng Hospital (Shanghai, China). All patients provided written informed consent, and articular cartilage samples were collected from the patients during hip arthroplasty surgery. All 20 patients with OA were diagnosed as grade IV according to the Kellgren & Lawrence (K-L) grading system. The 20 patients with femoral neck fractures had no history of hip pain and no evidence of OA on imaging examination. Two cartilage samples were collected from each femoral head. One sample was divided into two smaller pieces and frozen separately in liquid nitrogen for protein and RNA extraction. The other sample was immediately fixed in 4% paraformaldehyde overnight and embedded in paraffin for histological analyses. Samples of synovial fluids were taken and stored in liquid nitrogen until analyzed by IL-1β and TNF-α ELISA kits (R&D Systems). All measurements were performed in duplicate. No patients were invited to comment on the study design or contribute to the writing or editing of this manuscript.

### Animal experiments

Male mice on a C57BL/6 J background were purchased from Shanghai Slake Laboratory Animal Co., Ltd. All mice were maintained in a specific pathogen-free environment with food and water ad libitum. The mice were anesthetized with 2% isoflurane anesthesia, and the hindlimbs were shaved and prepared for aseptic surgery. For the DMM-induced OA mouse model, the medial meniscotibial ligament in the right knee joint was transected as previously described^14^. For the sham operation, the right knee joint was exposed following medial capsular incision, and the fat pad was dissected and then closed with sutures. For the therapeutic experiment, 1 day after surgery, the mice in the NR group were treated with an intraperitoneal injection of NR (1 mg/kg/day) in a total volume of 0.3 ml for 8 weeks, while mice in the control group (NC) received an equal volume of 0.4% DMSO. Pain behavioral analyses, including mechanical allodynia tests and spontaneous activity tests, were conducted 8 weeks after surgery. Mechanical allodynia was examined using von Frey filaments (North Coast Medical Inc., CA, USA). The mechanical allodynia test was performed using a calibrated set of von Frey filaments, which were applied to the right (ipsilateral) hind paw with graduated levels (typical force range was from 0.4 to 6.0 g). The paw sensitivity threshold was defined as the minimum pressure required to elicit a robust and immediate withdrawal reflex of the paw. Spontaneous behavior was measured by the Laboratory Animal Behavior Observation Registration and Analysis System (LABORAS). The LABORAS picked up vibrations due to animal movement and converted these into behavior classifications. Measurements were performed between 7:00 pm and 7:00 am the next day in recording cages. The following parameters were assessed: distance of locomotion, speed of locomotion, and rearing frequency. To generate *ATF5*^*f/f*^*Col2a1-CreER*^*T2*^ mice, *ATF5*^*f/f*^ mice were bred with a transgenic mouse line expressing Cre recombinase under the control of the type II collagen promoter and inducible by tamoxifen administration *(Col2a1-CreER*^*T2*^*)*. Before DMM surgery, tamoxifen (TM) (100 μg/g body weight, Sigma‒Aldrich) was intraperitoneally injected into 8-week-old *ATF5*^*f/f*^*Col2a1-CreER*^*T2*^ mice and their *ATF5*^*f/f*^ littermates daily for 5 days. Sham operations were performed on control mice. Eight weeks after DMM surgery, *ATF5*^*f/f*^*Col2a1-CreER*^*T2*^ mice and their *ATF5*^*f/f*^ littermates were killed for further analysis.

### Radiological evaluation

The knees of mice in the different groups were subjected to radiographical evaluation at the indicated time points. Briefly, digital plain radiographs of mouse knees were acquired with an MX-20 Cabinet X-ray System (Faxitron, Tucson, AZ, USA), and three-dimensional images were obtained with a SkyScan1172 high-resolution micro-CT system (Bruker, Kontich, Belgium) and supporting software.

### Histological analysis

Human and mouse cartilage samples were fixed with 4% paraformaldehyde overnight and then decalcified in a 10% EDTA solution for 30 days. After paraffin embedding, each sample was sectioned (5 μm thickness) and stained with H&E or safranin-O/fast green. For mouse samples stained with safranin-O/fast green, OARSI scores, synovitis scores, and osteophyte maturity were evaluated semiquantitatively as previously reported^15–17^.

### Immunohistochemistry and TUNEL staining

Paraffin-embedded cartilage samples were deparaffinized and rehydrated before further staining. For immunohistochemistry, the slides were incubated with primary antibodies against MMP-13, Col2a1, or Aggrecan at 4 °C overnight and treated with biotinylated secondary antibodies at 37 °C for 2 h. For TUNEL staining, a TUNEL Assay Kit (Roche, Switzerland) was used to stain the slides according to the manufacturer’s protocol.

### Primary mouse chondrocytes and synovial cell culture and treatment

Primary mouse chondrocytes were isolated from the knee joints of newborn mice as previously described^18^ and cultured in DMEM (Invitrogen, Carlsbad, CA, USA) supplemented with 10% fetal bovine serum (FBS). Primary mouse synovial cells were isolated from the synovial tissue of 4- to 6-week-old C57BL/6 J male mice. Briefly, synovial tissue of the knee joints was chopped into small pieces and digested with 0.2% type II collagenase and 0.45% trypsin for 8 h. After centrifugation, the sediment was resuspended and cultured in RPMI 1640 with 10% FBS, 100 U/mL penicillin, 100 µg/mL streptomycin, and 1% insulin-transferrin-selenium. Once the cells reached 80% confluence, the chondrocytes, and synovial cells were treated as indicated to examine whether the UPR^mt^ was activated in response to exposure to different pathological stresses: (1) inflammatory stress induced by IL-1β; (2) mitochondrial stress induced by the mitochondrial depolarizing agent valinomycin; and (3) mitochondrial stress induced by rotenone, an inhibitor of mitochondrial-respiration complex I.

For NR treatment, chondrocytes were pretreated with 1 mmol/l NR for 12 h and then cultured in DMEM/FBS containing 10 ng/ml IL-1β. To preserve the chondrocyte phenotype, the cells at less than two passages were used for in vitro experiments.

### Cell transfection

Atf5 siRNA plasmid vectors were designed and constructed. Primary mouse chondrocytes were transfected with 50 nM Atf5 siRNA plasmid vectors (si-Atf5) and negative control vectors (si-NC) using Lipofectamine 2000 (Invitrogen, Carlsbad, CA, USA) according to the manufacturer’s instructions. The cells were used for subsequent experiments 48 h after being transfected. The results of three independent experiments with duplicate wells were averaged.

### RNA extraction and quantitative real-time PCR

Total RNA was extracted from primary mouse chondrocytes and mouse and human cartilage samples using TRIzol reagent (Invitrogen, Carlsbad, CA, USA) according to the manufacturer’s protocol. Briefly, 50 mg of cartilage tissue was mechanically minced, frozen with liquid nitrogen, and ground into a fine powder. The RNA concentration was measured using a NanoDrop ND-1000 spectrophotometer (NanoDrop Technologies, DE, USA), and RNA integrity was assessed with an Agilent Bioanalyzer 2100 (Agilent Technologies, Santa Clara, CA, USA). RNA samples (1 μg) were then used for cDNA synthesis with a RevertAid First Strand cDNA Synthesis Kit (TaKaRa, Dalian, China), and the cDNA was amplified by qRT‒PCR using an SYBR Premix Ex Tag Kit (TaKaRa, Dalian, China) according to the manufacturer’s instructions. Each qPCR was performed three times with at least three different experimental replicates on an ABI 7500 Sequencing Detection System (Applied Biosystems, Foster City, CA, USA). GAPDH was used as an endogenous control. The data were analyzed by calculating the relative fold change using the following formula: 2 ‒ ΔΔCt. The primers used in this study are shown in Supplementary Table 1.

### Cell counting kit-8 (CCK-8) assay

The viability of primary mouse chondrocytes was assessed using the CCK-8 assay (Abcam, Cambridge, UK) according to the manufacturer’s instructions. Briefly, the cells were seeded into 96-well plates at a density of 5 × 10^3^ cells per well and cultured for the indicated times. After being incubated with 10 µl/well WST-8 solution for 1 h, the absorbance of each sample was measured at a wavelength of 460 nm.

### Cell proliferation assay

The proliferation of primary mouse chondrocytes was measured with a 5-ethynyl-2’-deoxyuridine (EdU) staining proliferation kit (Abcam, Cambridge, UK). Briefly, chondrocytes were seeded in 24-well plates at a density of 5 × 10^5^ cells per well and incubated with 50 μM EdU for 2 h. After being fixed and permeabilized, the cells were washed with PBS, incubated with 100 μl of the EdU reaction mixture per well for 30 min at room temperature, and then incubated with 100 µL of Hoechst 33258 for 30 min. Finally, the ratio of EdU-positive cells to total Hoechst 33342-positive cells was calculated.

### Flow cytometry

Primary mouse chondrocyte apoptosis was measured with an Annexin V-FITC Apoptosis Staining Kit (Abcam, Cambridge, UK). Briefly, chondrocytes were washed with PBS (4 °C) and collected with 0.25% trypsin. The cells (1 × 10^5^) were incubated with 500 µl of 1× Annexin V Binding Buffer and 5 μl of propidium iodide at room temperature for 5 min, and apoptotic cells were quantified by flow cytometry.

### Western blot analysis

Western blot analysis was performed according to standard methods. Briefly, total protein was extracted using RIPA buffer (Beyotime, Jiangsu, China). Because only a small amount of protein can be collected from the cartilage of an adult mouse, the cartilage tissues of 2–3 mice in the same group were combined into one sample and then subjected to further protein extraction. After being quantified by a BCA assay (Beyotime, Jiangsu, China), the protein samples were separated by SDS‒PAGE and transferred onto PVDF membranes. Then, the PVDF membranes were incubated with primary and secondary antibodies at the indicated dilutions. The primary antibodies used in this study were as follows: rabbit anti-Atf5 (1:2000, Abcam, ab184923), rabbit anti-MMP-13 (1:3000, Abcam, ab39012), rabbit anti-ADAMTS-5 (1:250, Abcam, ab41037), rabbit anti-Col2a1 (1:5000, Abcam, ab34712), mouse anti-Aggrecan (1:1000, Abcam, ab3778), rabbit anti-calreticulin (1:1000, CST, #12238), rabbit anti-Grp78 (1:1000, CST, #3177), and rabbit anti-Grp94 (1:1000, CST, #20292). A mouse anti-GAPDH antibody (1:10000, Abcam, ab8245) and rabbit anti-β-actin antibody (1:1000, CST, #8457) were used as internal controls, and an HRP-conjugated goat anti-rabbit antibody (1:10000, ASPEN, AS1107) was used as the secondary antibody. The protein bands were detected using the ECL method and processed using ImageJ software (NIH, Bethesda, MD, USA).

### Immunofluorescence and TUNEL staining

Primary mouse chondrocytes were rinsed in PBS and fixed with 4% paraformaldehyde for 15 min at room temperature. After being permeabilized with 0.3% Triton X-100 for 5 min, the cells were blocked with 1% BSA for 30 min and incubated with mouse anti-MMP-13 (1:300, Abcam, ab219620) and anti-Col2a1 (1:500, Abcam, ab34712) antibodies at 4 °C overnight. The samples were then washed with PBS (4 °C) three times and incubated with a Cy3-conjugated goat anti-rabbit secondary antibody (1:50, ASPEN, AS1109) at 37 °C for 30 min. TUNEL staining of chondrocytes was performed with a TUNEL Assay Kit (Roche, Basel, Switzerland) according to the manufacturer’s protocol.

### Mitochondrial respiratory complex activity measurement

The enzymatic activity of NADH ubiquinone reductase (complex I), succinate ubiquinone reductase (complex II), ubiquinol cytochrome C reductase (complex III), and cytochrome C oxidase (complex IV) in primary mouse chondrocytes were measured using enzyme activity assay kits (A089-1-A089-4, Nanjing Jiancheng Bioengineering Institute, China) according to the manufacturer’s instructions.

### Mitochondrial membrane potential measurement

JC‑1 staining was performed to measure the mitochondrial membrane potential. After the supernatants were incubated with JC-1 (0.5 μmol/L, Invitrogen) for 20 min at 37 °C, the samples were precipitated by centrifugation at 600 × *g* and 4 °C for 3 min. Primary mouse chondrocytes were washed twice with JC-1 staining buffer and centrifuged at 4 °C for 3 min to pellet the cells. The mitochondrial membrane potential was determined by measuring the ratio of aggregated JC-1 to monomeric JC-1.

### Transmission electron microscopy (TEM)

Primary mouse chondrocyte mitochondrial ultrastructure was analyzed by TEM. Cells were harvested and fixed with 2.5% glutaraldehyde and 2% osmium tetroxide in 0.1 M phosphate buffer (pH 7.4). After being dehydrated in an ascending ethanol series, the cells were embedded in Epon. Electron micrographs were obtained using a Zeiss EM10 electron microscope (Carl Zeiss AG).

### ELISA

Samples of synovial fluids were obtained from patients with hip OA and femoral neck fracture during hip arthroplasty surgery. ELISA (enzyme-linked immunosorbent assay) was used to measure IL-1β (#BMS224-2, Invitrogen, Carlsbad, CA, USA) and TNF-α (#BMS223-4, Invitrogen, Carlsbad, CA, USA) levels in synovial fluids according to the manufacturer’s instructions.

### Statistical analysis

All data are expressed as the means ± 95% CI, and all statistical analyses were performed using GraphPad Prism software (version 7.0). The Kolmogorov‒Smirnov test was used to confirm the normal distribution of the data. Statistical significance was evaluated by Student’s paired and unpaired *t* tests for parametric data or the Mann‒Whitney *U* test and Wilcoxon matched-pairs signed rank test for nonparametric data. ANOVA was used to compare more than two groups. For all analyses, *p* < 0.05 was considered significantly different.

## Results

### UPR^mt^ induction in primary mouse chondrocytes and in the cartilage of OA mice

**Fig. 1:**
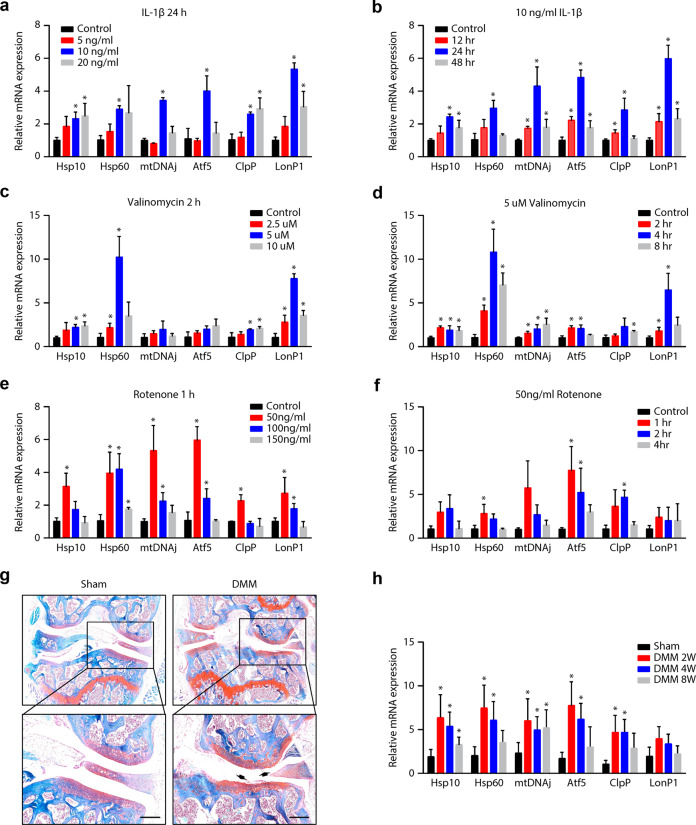
Induction of the UPR^mt^ in stressed primary mouse chondrocytes and the cartilage of OA mice. **a**, **b** Response of chondrocytes to IL-1β (5, 10, or 20 ng/ml for 24 h or 10 ng/ml for 12, 24, or 48 h). **c**, **d** Response of chondrocytes to valinomycin (2.5, 5, or 7.5 μM for 2 h or 5 μM for 2, 4, or 6 h). **e**, **f** Response of chondrocytes to rotenone (50, 100, or 150 ng/ml for 1 h or 50 ng/ml for 1, 2, or 4 h). *p < 0.05, differences in the mRNA levels of UPR^mt^ markers compared with those in the control group; *n* = 3. **g** Representative images of mouse knee joints stained with safranin-O/fast green in the sham group and DMM surgery group (8 weeks after surgery). Black arrows indicate articular cartilage degradation; scale bar, 100 mm. **h** Expression of UPR^mt^ markers in the cartilage of DMM-induced OA mice; *p < 0.05 compared with the sham group; *n* = 6. Hsp10 = Heat shock 10 kDa protein 1; Hsp60 = Heat shock 60 kDa protein 1; mtDNAj = mitochondrial presequence translocase-associated motor complex protein; Atf5 = cyclic AMP-dependent transcription factor ATF5; ClpP = ATP-dependent Clp protease proteolytic subunit; LonP1 = Lon protease homolog; UPR^mt^ = mitochondrial unfolded protein response.

Primary mouse chondrocytes treated with IL-1β, rotenone and valinomycin showed time- and concentration-dependent upregulation of the mRNA expression of Hsp10, Hsp60, ClpP, LonP1, mtDNAj, and Atf5 (Fig. 1a–f). In addition, we found that the increases in the mRNA levels of mitochondrial chaperones and proteases were unimodal following exposure to different stresses. For instance, the mRNA levels of UPR^mt^ markers were elevated after IL-1β treatment and peaked after treatment with 10 ng/ml IL-1β for 24 h (Fig. 1a, b); similarly, the upregulation of UPR^mt^ marker expression induced by valinomycin peaked in response to 5 μM valinomycin treatment for 2 h (Fig. 1c, d), while rotenone treatment increased the expression of UPR^mt^ markers at an earlier stage or at a lower concentration (Fig. 1e, f). To investigate whether the UPR^mt^ was also induced in synovial cells by these stimuli, primary mouse synovial cells were isolated and treated with IL-1β, rotenone, and valinomycin. Interestingly, the mRNA levels of mitochondrial chaperones and proteases remained unchanged in response to the different stress conditions (Supplementary Fig. 1a–c), indicating a specific effect of these stimuli on the induction of the UPR^mt^ in chondrocytes.

A previous study indicated that the UPR^mt^ was distinct from cytosolic stress responses and the endoplasmic reticulum unfolded protein response (ER stress)^13^. To assess this in chondrocytes, the transcriptional levels of cytosolic chaperones (Hsp70 and Hsp90) and the protein levels of ER stress markers (calreticulin, Grp78 and Grp79) were determined under these pathological conditions. The results showed that the mRNA levels of cytosolic Hsp70 and Hsp90 were unaltered in chondrocytes treated with IL-1β, valinomycin and rotenone (Supplementary Fig. 1d–f). Similarly, the protein levels of Grp78 and Grp79 were also unaffected by these UPR^mt^-inducing stresses (Supplementary Fig. 1g).

We next investigated whether the UPR^mt^ was activated in OA cartilage tissue. DMM surgery was performed to induce an in vivo model of OA, and the successful establishment of the OA model was confirmed by typical morphological changes of severe articular destruction and loss of integrity (Fig. 1g). Furthermore, the PCR results showed that the changes in the expression of UPR^mt^ markers showed a unimodal trend in cartilage tissues, peaking at 2 weeks after DMM surgery and gradually decreasing at 4 and 8 weeks (Fig. 1h). Collectively, these data demonstrated for the first time that the UPR^mt^ was induced in chondrocytes following exposure to different pathological stresses and in the articular cartilage tissue of OA mice.

### Enhancing the UPR^mt^ improves mitochondrial function in stressed primary mouse chondrocytes

**Fig. 2:**
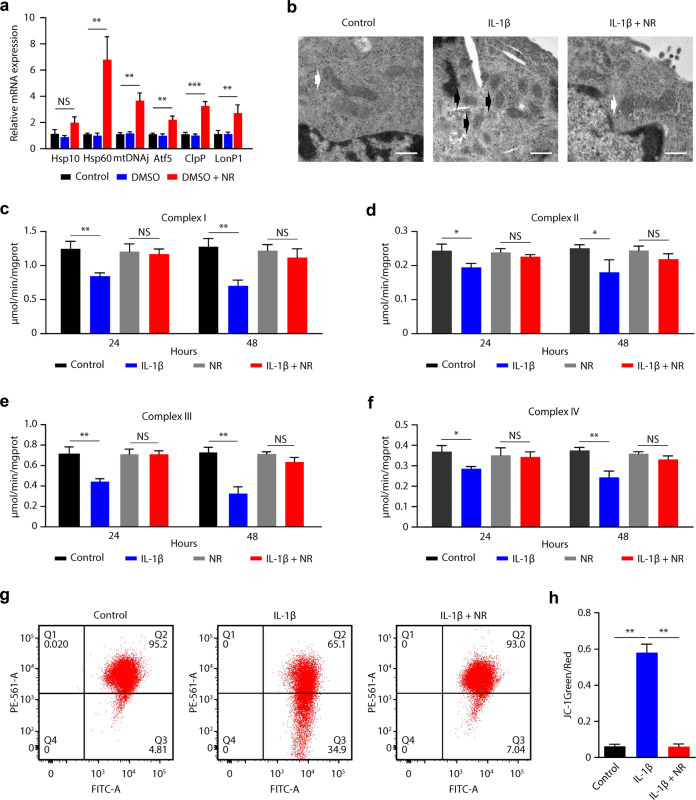
Enhancing the UPR^mt^ improves mitochondrial function in stressed primary mouse chondrocytes. **a** Compared with control chondrocytes, chondrocytes treated with NR (1 mmol/l) showed increased mRNA levels of Hsp10, Hsp60, mtDNAj, Atf5, ClpP, and LonP1. **p < 0.01, ***p < 0.001; NS = not significant; *n* = 3. **b** Electron micrographs of chondrocytes showing intact mitochondria (white arrow, control, and IL-1β + NR groups) with well-preserved double membranes and cristae structures and fragmented mitochondria (black arrow, IL-1β group) with deformed or absent cristae structures. Scale bar, 500 nm. **c**–**f** Mitochondrial respiratory chain complex I–IV activity in chondrocytes treated with IL-1β. *p < 0.05, **p < 0.01 compared with the control group; NS = not significant; *n* = 3. **g** Flow cytometric analysis of JC‑1 distribution in chondrocytes. **h** The JC-1 ratio was calculated as the number of green JC-1-positive mitochondria relative to the number of red JC-1-positive mitochondria. **p < 0.01; *n* = 3.

Previous studies have shown that supplementation with nicotinamide adenine dinucleotide (NAD+) precursors such as NR enhances the UPR^mt ^^19^. Consistent with these data, the mRNA expression of Hsp10, Hsp60, ClpP, LonP1, mtDNAj, and Atf5 was significantly upregulated in chondrocytes after treatment with NR (1 mmol/l for 12 h) (Fig. 2a), while the expression levels of cytosolic chaperones and ER stress proteins remained unchanged (Supplementary Fig. 2a, b).

To determine the effects of enhancing the UPR^mt^ on stressed primary mouse chondrocytes, we assessed the effects of NR treatment on mitochondrial morphology, mitochondrial respiratory function, and mitochondrial membrane potential. We evaluated the effect of IL-1β, the most important inflammatory cytokine and one that is frequently used as a stressor in OA research, on mitochondrial morphology in chondrocytes. Compared with mitochondria in the control group, IL-1β treatment induced mitochondrial fragmentation with disorganization of cristae structure. However, NR pretreatment protected mitochondria, which exhibited well-preserved double membranes and cristae structures (Fig. 2b). We then tested the effects of the UPR^mt^ on mitochondrial respiratory function by assessing the activity of respiratory chain complexes I-IV. We found that IL-1β significantly inhibited mitochondrial complex I-IV activity in chondrocytes and that this effect was alleviated by NR pretreatment (Fig. 2c–f). Mitochondrial membrane potential was also measured by the JC-1 assay, and the results demonstrated that the mitochondrial membrane potential in the IL-1β-treated chondrocytes was reduced compared with that in the control group; however, treatment with NR significantly improved the mitochondrial membrane potential of chondrocytes subjected to IL-1β treatment (Fig. 2g, h). These data further demonstrated that IL-1β caused mitochondrial damage in chondrocytes and that enhancing the UPR^mt^ with NR improved mitochondrial function in stressed chondrocytes.

### Enhancement of the UPR^mt^ is cytoprotective in stressed primary mouse chondrocytes

**Fig. 3:**
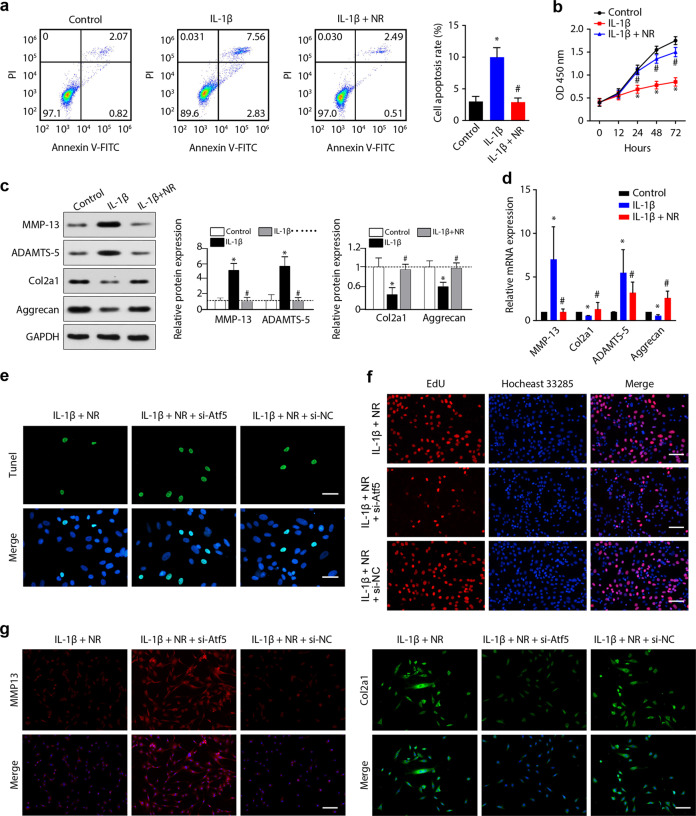
Enhancing the UPR^mt^ with NR is cytoprotective in chondrocytes treated with IL-1β. **a** The effect of NR on cell apoptosis was analyzed by flow cytometry, and the apoptosis rate was calculated. *p < 0.05 compared with the control group; ^#^*p* < 0.05 compared with the IL-1β group; *n* = 3. **b** The OD 450 values of chondrocytes in the IL-1β group were dramatically lower than those in the control group and were significantly increased by NR. *p < 0.05 compared with the control group; ^#^*p* < 0.05 compared with the IL-1β group; *n* = 3. **c** Western blot analysis of MMP-13, ADAMTS-5, Col2a1 and Aggrecan expression in chondrocytes in the different groups. *p < 0.05 compared with the control group; ^#^*p* < 0.05 compared with the IL-1β group; *n* = 3. **d** qPCR analysis of MMP-13, ADAMTS-5, Col2a1, and Aggrecan expression in chondrocytes in the different groups. *p < 0.05 compared with the control group; ^#^*p* < 0.05 compared with the IL-1β group; *n* = 3. **e** TUNEL analysis of chondrocyte apoptosis. Green fluorescence indicates apoptotic cells, and blue fluorescence indicates normal chondrocytes. Scale bar, 50 μm. **f** Cell proliferation in vitro was assessed by the EdU assay. Scale bar, 50 μm. **g** Immunofluorescence staining of MMP-13 and Col2a1 in chondrocytes in the different groups. Scale bar, 50 μm.

To determine the role of the UPR^mt^ in the pathological process of OA, we further investigated the effects of UPR^mt^ enhancement on chondrocyte apoptosis, proliferation, and ECM metabolism. The flow cytometry results revealed that the increase in the apoptosis rate induced by IL-1β was markedly suppressed by NR treatment (Fig. 3a). The CCK-8 results showed that the inhibition of chondrocyte proliferation in response to IL-1β treatment was significantly alleviated by NR (Fig. 3b). Additionally, as shown by western blotting and PCR analysis, IL-1β treatment decreased the synthesis of cartilage ECM components (Aggrecan and Col2a1) and increased the secretion of cartilage-degrading enzymes (MMP-13 and ADAMTS-5) at both the transcriptional and protein levels, and these changes were ameliorated by NR treatment (Fig. 3c, d).

Atf5 has been reported to play a key role in inducing the expression of Hsp10, Hsp60, ClpP, LonP1, and mtDNAj following UPR^mt^ activation in mammals^11^, and we found that silencing Atf5 in primary mouse chondrocytes abolished the IL-1β-induced upregulation of UPR^mt^ markers (Supplementary Fig. 3a). To confirm that the effects of NR are mediated by the activation of the UPR^mt^, we silenced Atf5 and then examined the effects of NR on chondrocyte apoptosis, proliferation, and ECM metabolism by TUNEL staining, EdU assays, and immunofluorescence assays. The results showed that silencing Atf5 substantially suppressed the NR-induced increases in UPR^mt^ markers (Supplementary Fig. 3a). In addition, silencing Atf5 inhibited the NR-mediated antiapoptotic, proliferative, proanabolic, and anticatabolic effects of IL-1β treatment (Fig. 3e–g, Supplementary Fig. 3b–e). Together, these data clearly indicated that enhancing the UPR^mt^ by NR ameliorated the detrimental effects of IL-1β on chondrocyte proliferation, apoptosis, and ECM metabolism.

### Enhancing the UPR^mt^ with NR ameliorates DMM-induced OA and attenuates OA pain

**Fig. 4:**
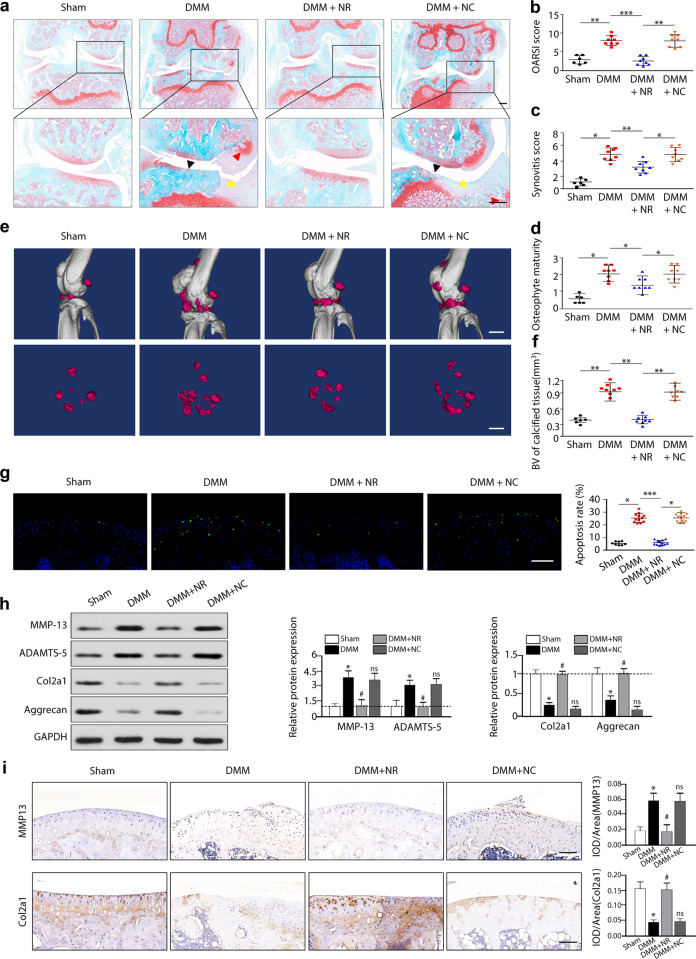
Enhancing the UPR^mt^ with NR ameliorates DMM-induced OA in vivo. **a** Safranin-O/fast green staining of the cartilage of mice in the different groups. Black arrowheads indicate articular cartilage degradation, yellow arrowheads indicate synovial hyperplasia, and red arrowheads indicate osteophytes. Scale bar, 200 μm. **b** Quantitative analysis of the OARSI score, **c** synovitis score, and **d** osteophyte maturity in the different groups of mice. *p < 0.05, **p < 0.01, ***p < 0.001; *n* = 6–8. **e** Micro-CT imaging of the morphological structure of the knee in mice in the different groups. Scale bar, 10 mm. **f** The volumes of the calcified meniscus and synovial tissue were quantified. **p < 0.01; *n* = 6–8. **g** TUNEL analysis of chondrocyte apoptosis in mouse knee cartilage. Green fluorescence indicates apoptotic cells, and blue fluorescence indicates normal chondrocytes. Scale bar, 100 μm; *p < 0.05, ***p < 0.001; *n* = 8–12. **h** Western blot analysis of MMP-13, Col2a1, ADAMTS-5, and Aggrecan expression in knee articular cartilage in the different groups. *p < 0.05 compared with the sham group; ^#^*p* < 0.05 compared with the DMM group; NS not significant compared with the DMM group; *n* = 8–12. **i** Immunohistochemical staining of MMP-13 and Col2a1 in knee articular cartilage in the different groups. Scale bar, 100 μm; *p < 0.05 compared with the sham group; ^#^*p* < 0.05 compared with the DMM group; NS not significant compared with the DMM group; *n* = 6.

To further investigate the effect of UPR^mt^ enhancement on OA in vivo, mice were treated with NR or DMSO for 8 weeks after DMM surgery. Systemic treatment with NR successfully increased the expression of Hsp10, Hsp60, mtDNAj, Atf5, ClpP, and LonP1 in cartilage tissue in mice that underwent DMM surgery (Supplementary Fig. 4a), while treatment with NR did not affect the cytosolic stress responses or ER stress response (Supplementary Fig. 4b, c). Histological analyses were used to assess knee joint damage, including articular cartilage degradation, synovial tissue hyperplasia, and osteophyte formation (Fig. 4a). Assessment of articular cartilage degradation by OARSI scores revealed that DMM surgery caused severe cartilage degeneration, as evidenced by markedly increased OARSI scores compared with controls, and the administration of NR significantly alleviated cartilage degradation (Fig. 4b). Synovial tissue hyperplasia and osteophyte formation were also analyzed by determining the synovitis score (Fig. 4c) and osteophyte maturity (Fig. 4d), and the results showed that NR treatment significantly inhibited synovitis and osteophyte formation in mice with OA induced by DMM surgery. In addition, micro-CT analysis revealed a significant increase in osteophyte formation in DMM-induced mice, and this effect was significantly inhibited by boosting the UPR^mt^ with NR treatment (Fig. 4e, f).

Furthermore, we determined the role of the UPR^mt^ in chondrocyte apoptosis and ECM metabolism in DMM-induced OA mice. Histological analysis of cartilage tissue revealed that NR treatment significantly reduced the number of TUNEL + chondrocytes in OA mice induced by DMM surgery (Fig. 4g). In addition, as evidenced by western blotting and immunohistochemistry, enhancing the UPR^mt^ maintained the balance between cartilage ECM anabolism and catabolism, decreasing cartilage-degrading enzyme secretion and enhancing ECM synthesis (Fig. 4h-i). These data demonstrated that enhancing the UPR^mt^ improved chondrocyte survival, decreased OARSI scores, and maintained the balance between cartilage ECM anabolism and catabolism in DMM-induced OA model mice.

To determine whether enhancing the UPR^mt^ inhibited the increased pain sensitivity caused by DMM surgery, we performed a series of pain behavioral analysis measurements, including mechanical allodynia tests and spontaneous activity tests, as assessed by the Laboratory Animal Behavior Observation Registration and Analysis System (LABORAS). The results of the mechanical allodynia test showed that paw withdrawal response thresholds were significantly reduced after DMM surgery in mice without NR treatment. The administration of NR significantly increased the paw withdrawal response thresholds (Supplementary Fig. 4d). In addition, we found that the locomotion distance, locomotion speed, and rearing frequency were also significantly reduced in mice that underwent DMM surgery without NR treatment. NR significantly reversed the reduction in spontaneous activity caused by DMM surgery (Supplementary Fig. 4e–g), indicating that NR treatment could attenuate OA pain.

### Conditional knockout of Atf5 in chondrocytes reduced the protective effects of NR against OA progression

**Fig. 5:**
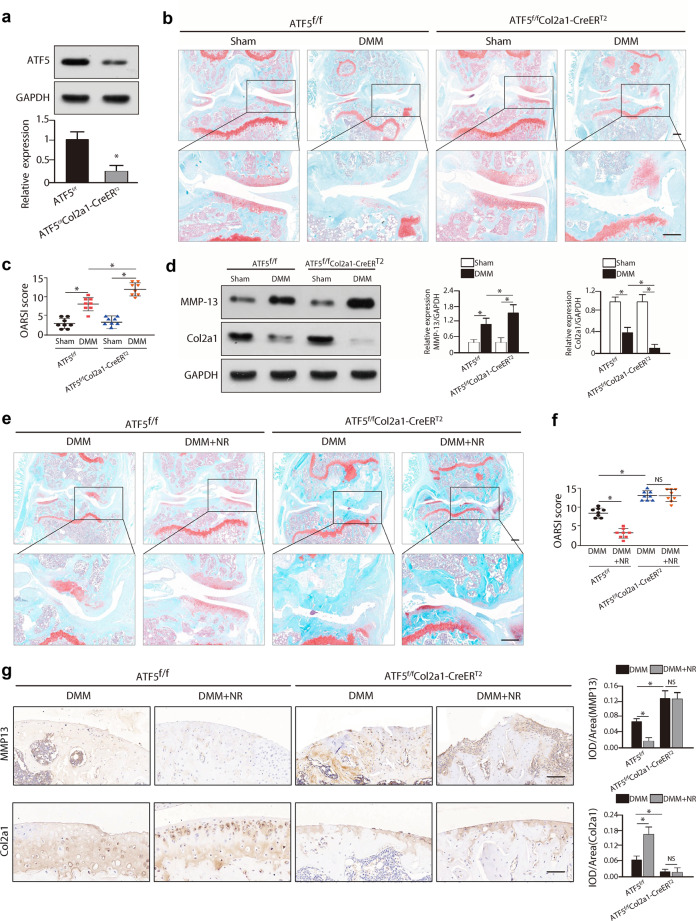
Conditional knockout of Atf5 in chondrocytes reduced the protective effects of NR against OA progression. **a** Western blot analysis of Atf5 in the knee joint cartilage of *ATF5*^*f/f*^*Col2a1-CreER*^*T2*^ mice and *ATF5*^*f/f*^ mice. *p < 0.05; *n* = 6. **b** Safranin-O/fast green staining of the cartilage of *ATF5*^*f/f*^*Col2a1-CreER*^*T2*^ mice and *ATF5*^*f/f*^ mice. Scale bar, 200 μm. **c** Quantitative analysis of the OARSI scores in different groups of mice. *p < 0.05; *n* = 8. **d** Western blot analysis of MMP-13 and Col2a1 in the cartilage of *ATF5*^*f/f*^*Col2a1-CreER*^*T2*^ mice and *ATF5*^*f/f*^ mice. *p < 0.05; *n* = 6. **e** Safranin-O/fast green staining of the cartilage of *ATF5*^*f/f*^*Col2a1-CreER*^*T2*^ mice and *ATF5*^*f/f*^ mice with or without NR treatment following DMM surgery. Scale bar, 200 μm. **f** The OARSI scores in the different groups. *p < 0.05; NS = not significant; *n* = 6. **g** Immunohistochemical staining of MMP-13 and Col2a1 in cartilage in the different groups. Scale bar, 100 μm. *p < 0.05; NS = not significant; *n* = 6.

To determine the role of Atf5 in UPR^mt^-mediated OA progression, we generated *ATF5*^*f/f*^*Col2a1-CreER*^*T2*^ mice as described in the Methods Section. Knockout efficiency was confirmed by Western blot analysis of ATF5 in the knee joint cartilage (Fig. 5a), and the PCR results demonstrated that knockout of Atf5 in mice significantly downregulated the mRNA expression of UPR^mt^ markers (Supplementary Fig. 5a). Compared with *ATF5*^*f/f*^ control mice, *ATF5*^*f/f*^*Col2a1-CreER*^*T2*^ mice exhibited more severe DMM-induced cartilage degeneration at 8 weeks after surgery (Fig. 5b, c). In addition, the expression levels of the cartilage ECM component Col2a1 and cartilage-degrading enzyme MMP-13 were decreased and increased, respectively, in conditional knockout mice after DMM surgery, further demonstrating the severe progression of OA after Atf5 knockout (Fig. 5d).

Furthermore, to investigate whether Atf5 plays a critical role in NR-mediated enhancement of the UPR^mt^ during OA, *ATF5*^*f/f*^*Col2a1-CreER*^*T2*^ mice were treated with or without NR for 8 weeks after DMM surgery. The PCR results showed that NR treatment significantly enhanced DMM-induced UPR^mt^ activation, while the mRNA expression of UPR^mt^ markers remained unchanged in *ATF5*^*f/f*^*Col2a1-CreER*^*T2*^ mice after NR treatment (Supplementary Fig. 5b). As evidenced by safranin-O/fast green staining and OARSI scores, boosting the UPR^mt^ with NR treatment alleviated DMM-induced cartilage degeneration, but there were no significant differences in cartilage degeneration in DMM-induced *ATF5*^*f/f*^*Col2a1-CreER*^*T2*^ mice treated with or without NR (Fig. 5e, f). In addition, immunohistochemical staining of cartilage tissue confirmed that there was no difference in Col2a1 or MMP-13 expression in the cartilage tissues of Atf5 knockout mice treated with or without NR (Fig. 5g). Taken together, these results showed that the loss of Atf5 accelerated DMM-induced OA development, and the protective effects of NR against OA were dependent on the enhancement of the UPR^mt^ by targeting Atf5.

### Induction of the UPR^mt^ in cartilage tissues from OA patients

To investigate the relevance of the UPR^mt^ in OA patients, we analyzed cartilage tissues from 20 patients with grade IV hip OA according to the Kellgren & Lawrence (K-L) grading system^20^ who underwent hip replacement surgery, and control cartilage tissues were obtained from 20 patients with femoral neck fracture who underwent hip replacement surgery (Supplementary Fig. 6a). The clinical and epidemiological characteristics of these patients are shown in Supplementary Table 2.

**Fig. 6:**
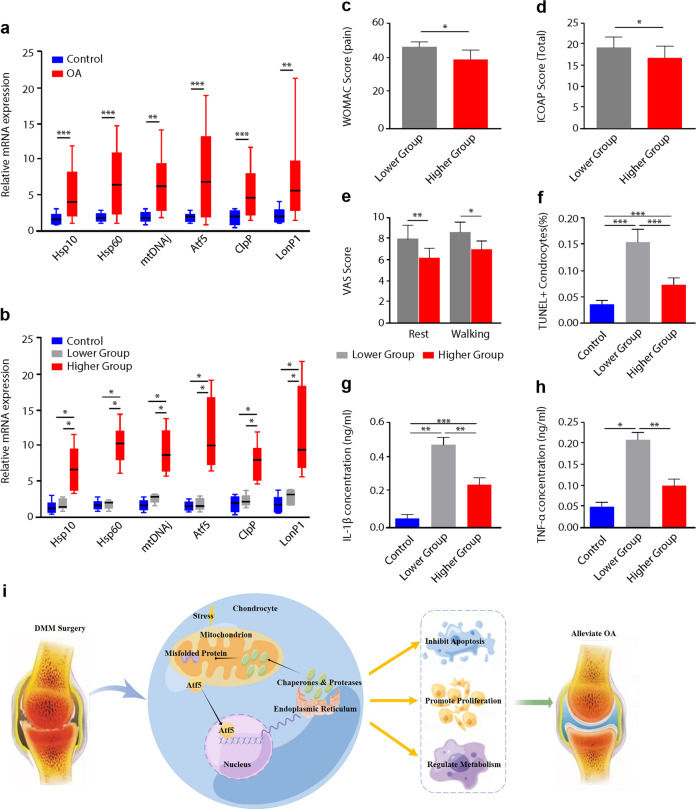
Induction of the UPR^mt^ in cartilage tissues from OA patients. **a** qPCR analysis of the expression of UPR^mt^ markers in the cartilage tissues of patients in the different groups. *p < 0.05, **p < 0.01, ***p < 0.001; *n* = 20. **b** Patients were divided into two subgroups based on the mRNA levels of UPR^mt^ markers that were higher or lower than 2 SDs from the mean for each transcript in the control group, and mRNA levels of UPR^mt^ markers in the different groups are shown. *p < 0.05, **p < 0.01, ***p < 0.001; *n* = 9–20. **c**–**e** Comparison of the clinical characteristics between OA patients with lower or higher UPR^mt^ markers. **c** WOMAC scores of pain. **d** Total ICOAP scores. **e** VAS scores at rest and walking; *p < 0.05, *n* = 9–11. **f**–**h** Measurement of chondrocyte apoptosis and inflammation levels in the cartilage tissue and synovial fluid of OA patients with lower or higher UPR^mt^ marker levels. **f** TUNEL + chondrocytes, **g** IL-1β levels, and (**h**) TNF-α levels. *p < 0.05, **p < 0.01, ***p < 0.001; *n* = 9–11. **i** Schematic of the hypothesized mechanism of the UPR^mt^ in OA.

The results confirmed that degenerative changes, such as increased expression of MMP-13 and ADAMTS-5 and decreased expression of Aggrecan and Col2a1, occurred in the cartilage matrix of OA patients (Supplementary Fig. 6b, c). Consistent with the data from DMM-induced OA model mice, the expression levels of UPR^mt^ markers were significantly increased in the cartilage from OA patients compared with controls (Fig. 6a). However, the mRNA levels of UPR^mt^ markers varied greatly among the 20 OA patients. Approximately 11 patients had significant increases in the expression levels of UPR^mt^ markers. To further determine the relationship between the expression levels of UPR^mt^ markers and the clinical characteristics of patients, we divided the OA patients into two groups: (1) OA patients with higher UPR^mt^ levels and (2) OA patients with lower UPR^mt^ levels. We divided the OA patients according to the mRNA levels UPR^mt^ markers that were higher or lower than 2 SDs from the mean for each transcript in the control group. OA patients with higher UPR^mt^ levels exhibited significant increases in all UPR^mt^ markers compared with those in the control group, indicating significant activation of the UPR^mt^ in this group (Fig. 6b). We compared the severity of hip pain at rest and during walking on visual analog scales (VAS, 0=no pain, 10=severe pain) for pain during the previous week and examined the patients with the Western Ontario and McMaster University Osteoarthritis Index scale (WOMAC)^21^ and Intermittent and Constant Osteoarthritis Pain (ICOAP)^22^. We also recorded patient medical consumption, such as analgesic use, before surgery. Histological analysis of cartilage tissue and ELISA analysis of synovial fluid were also performed on the two groups. There was no significant difference in age, sex, body mass index, the duration of hip OA symptoms, or frequent pain medical consumption between the two groups (Supplementary Table 2). Similar function and stiffness were identified in the two groups, as assessed by the WOMAC (Supplementary Fig. 6d, e), and OA patients with increased UPR^mt^ levels had less hip pain based on WOMAC assessment (Fig. 6c). Similar results were observed after comparing the ICOAP (Fig. 6d and Supplementary Fig. 6f) and VAS scores (Fig. 6e) between the two groups. Moreover, OA patients with higher UPR^mt^ levels had significantly fewer TUNEL + chondrocytes (Fig. 6f) in cartilage tissue and lower expression of IL-1β and TNF-α in synovial fluid (Fig. 6g, h).

Taken together, these data demonstrated that the UPR^mt^ was induced in human cartilage tissues during OA progression and was involved in the pathological process of OA. A high degree of UPR^mt^ activation was associated with reduced chondrocyte death, less severe hip pain, and lower levels of inflammation in synovial fluid, exerting a protective effect against OA and suggesting a potential target for OA therapy.

## Discussion

Normal mitochondria are required for numerous cellular functions, such as ATP generation and cell death regulation. Mitochondrial homeostasis is maintained through different mitochondrial stress responses, including mitophagy^23^, retrograde signaling^24^, and the UPR^mt ^^25^. The UPR^mt^ was identified and comprehensively characterized in *C. elegans* and later identified in other organisms, such as yeast^26^ and flies^27^. However, the role of the UPR^mt^ in mammalian cells has not been extensively studied. In particular, the role of the UPR^mt^ in chondrocytes in OA is unclear. Our study identified a protective role for the UPR^mt^ in chondrocytes in OA, and enhancing the UPR^mt^ improved mitochondrial function and ameliorated OA.

Although the UPR^mt^ was first discovered in mammalian cells^10^, its activation and role in *C. elegans* are better understood^28^. In *C. elegans*, the transcription factor Atf-1, which regulates metabolism and supports the recovery of mitochondrial function under stress, is a key regulator of the UPR^mt ^^29^. Similarly, in mammalian cells, the functional ortholog of Atf-1, Atf5, is a bZIP transcription factor that is shuttled from mitochondria to the nucleus. Under mitochondrial stress, Atf5 induces the expression of mitochondrial chaperones such as Hsp10, Hsp60, and mtDNAj and the mitochondrial proteases ClpP and LonP1^28,30,31^. In the present study, activation of the UPR^mt^ was observed in mouse chondrocytes under different pathological stresses in vitro and in the articular cartilage tissues of DMM-induced OA mice and OA patients. Interestingly, the expression of UPR^mt^ markers showed a unimodal trend in chondrocytes subjected to different pathological stresses, and the upregulation of UPR^mt^ markers in articular cartilage tissues peaked at 2 weeks after DMM surgery, which was much earlier than the occurrence of morphological changes in articular cartilage, indicating that the induction of the UPR^mt^ played critical roles in an early stage of OA, and UPR^mt^ markers might be potential targets to diagnose early OA.

The UPR^mt^ is activated to preserve mitochondrial proteostasis by inducing the mitochondrial protein quality control system, including chaperones and proteases. Consequently, enhancing the UPR^mt^ has been hypothesized to be cytoprotective under mitochondrial stress conditions. Increasing NAD(+) levels has been shown to induce the UPR^mt^ and exert protective effects under mitochondrial stress conditions. Mouchiroud et al.^32^ showed that restoration of NAD(+) levels activated the UPR^mt^ to prevent age-associated metabolic decline and promote longevity in worms. Ioannis et al.^13^ found that enhancing the UPR^mt^ with NR led to significant improvements in mitochondrial function in stressed cardiomyocytes. Similarly, we found that enhancing the UPR^mt^ with NR ameliorated DMM-induced OA and attenuated OA pain, as evidenced by improved chondrocyte survival and decreased OARSI scores. In addition, NR maintained the balance between cartilage ECM anabolism and catabolism and reversed the reduction in spontaneous activity in DMM-induced OA model mice. To further determine whether the protective effect of NR was dependent on UPR^mt^ activation, we silenced Atf5 in IL-1β-treated chondrocytes and found inhibition of NR-mediated antiapoptotic, proliferative, proanabolic, and anticatabolic effects. We then generated *ATF5*^*f/f*^*Col2a1-CreER*^*T2*^ mice and found more severe DMM-induced cartilage degeneration and increased osteophyte formation compared with controls. Interestingly, there were no significant differences in the severity of OA between *ATF5*^*f/f*^*Col2a1-CreER*^*T2*^ mice treated with or without NR and subjected to DMM surgery, suggesting that the protective effects of NR on OA are dependent on enhancing the UPR^mt^ by targeting Atf5. Taken together, these data showed that NR exerted cytoprotective effects on chondrocytes through UPR^mt^ enhancement instead of other NAD(+)-dependent mechanisms. NR treatment significantly ameliorated DMM-induced cartilage destruction, inhibited chondrocyte apoptosis and decreased the progression of OA features. Moreover, the effect of DMM surgery on the expression of MMP-13, ADAMTS-5, Col2a1 and Aggrecan was reversed by NR treatment. These data showed that the UPR^mt^ in chondrocytes may be a potential therapeutic target for OA. In addition, according to the unimodal trend in UPR^mt^ upregulation, we could infer that UPR^mt^ markers were upregulated at an early stage of OA to prevent pathological progression. However, as the disease progressed, multiple factors, such as inflammation and reactive oxygen species, result in severe protein misfolding that exceeds the capability of the UPR^mt^ and impairs mitochondrial function. All these effects lead to insufficient activation of the UPR^mt^ and a progressive pathological process. Therefore, when the UPR^mt^ was enhanced by NR in chondrocytes, mitochondrial function was improved, and OA progression was prevented.

Finally, to further translate our findings to the clinic, we examined cartilage tissues and synovial fluid from patients with OA and patients with femoral neck fractures undergoing hip replacement. The expression of UPR^mt^ markers in cartilage tissue was elevated in OA patients compared to healthy controls, which was consistent with the experimental evidence. A comprehensive histological analysis of cartilage tissue, the severity of hip pain, medical consumption, and inflammation in synovial fluid indicated a close relationship between enhanced activation of the UPR^mt^ and reduced chondrocyte damage. We found that OA patients who had higher levels of the UPR^mt^ had a lower degree of pain. They also showed lower levels of inflammation in synovial fluid and fewer apoptotic chondrocytes in cartilage tissue, which are both important pathogenic factors of OA. These data show the activation of the UPR^mt^ in OA for the first time and suggest that enhancing the UPR^mt^ may be a novel therapeutic strategy for OA.

In conclusion, the current study revealed that the UPR^mt^ was induced in chondrocytes subjected to different pathological stresses and in the articular cartilage of OA model mice. Enhancing the UPR^mt^ with NR improved mitochondrial function and ameliorated OA. In addition, the cartilage tissue from patients with OA also showed evidence of UPR^mt^ activation, which was associated with reduced chondrocyte death, less severe hip pain, and lower levels of inflammation in synovial fluid. These findings reveal an important protective role for the UPR^mt^ in the progression of OA and provide a potential diagnostic and therapeutic strategy for OA (Fig. 6i). However, more studies are needed to investigate the kinetics of the UPR^mt^ activation in chondrocytes and better elucidate the therapeutic potential of NR in OA. In addition, further randomized clinical trials will help to determine the clinical effects of enhancing UPR^mt^ activation in the chondrocytes of OA patients.

## Supplementary information

SUPPLEMENTAL MATERIAL
