# Supplementary material for: The mitochondrial unfolded protein response (UPRmt) protects against osteoarthritis
Source: Exp Mol Med. 2022 Nov 15;54(11):1979–90. doi: 10.1038/s12276-022-00885-y (PMC9723171; doi:10.1038/s12276-022-00885-y)
Supplement: Supplementary file 1 — SUPPLEMENTAL MATERIAL [file 12276_2022_885_MOESM1_ESM.pdf]

Supplementary Figures

Supplementary Fig. 1. UPR^{mt} is not induced in synovial cells, and is a process distinct from ER stress response and cytosolic stress response in primary mice chondrocytes.
(**a-c**) Response of synovial cells to IL-1β (**a**), valinomycin (**b**) and rotenone (**c**). (**d-f**) mRNA levels of the cytosolic chaperones Hsp70 and Hsp90 in chondrocytes treated with IL-1β (**d**), valinomycin (**e**) and rotenone (**f**). *$p<0.05$ compared with the control group; n=3. (**g**) ER stress proteins levels (i.e. calreticulin, Grp78 and Grp94) after treatment with IL-1β, rotenone and valinomycin.

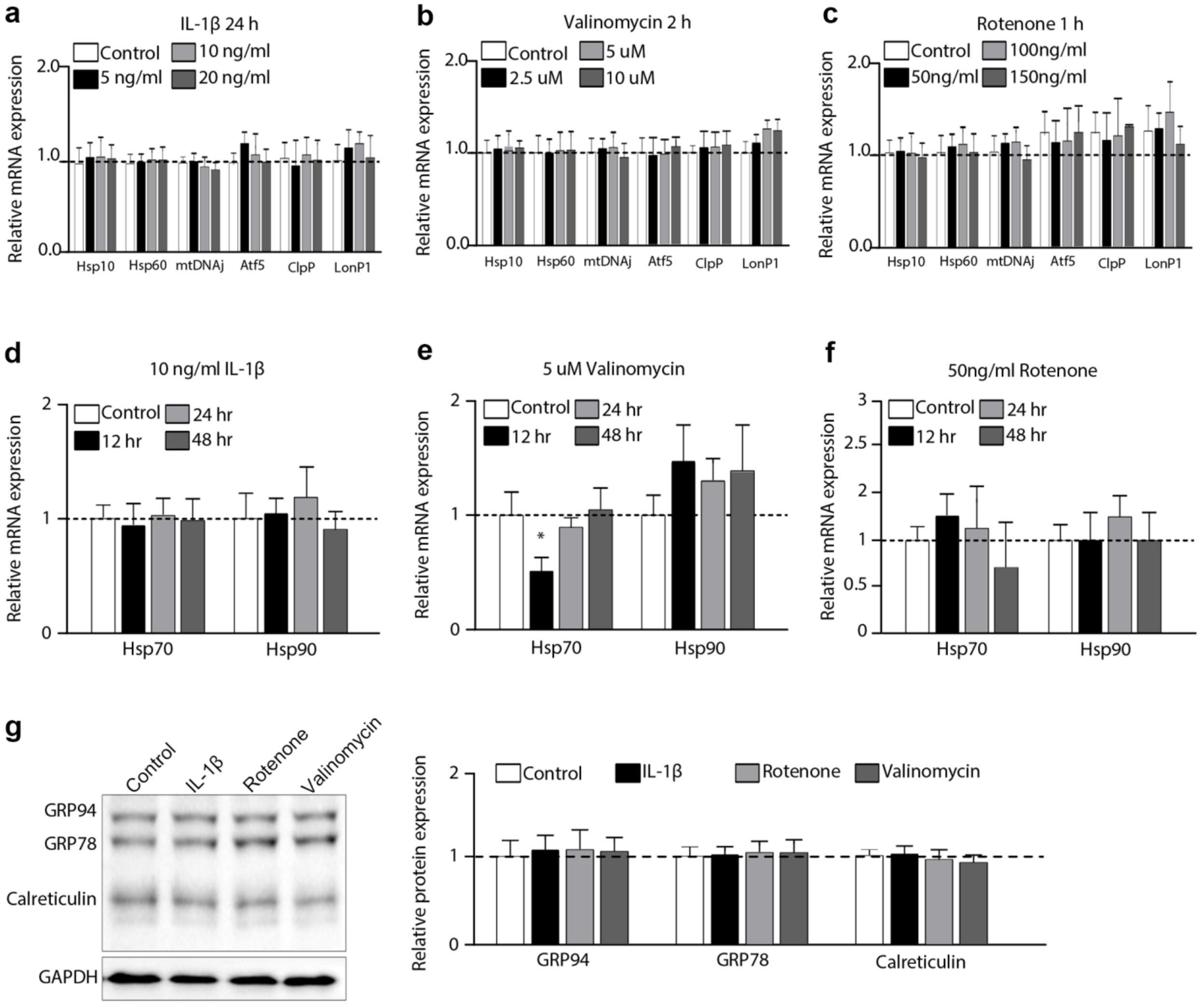

Supplementary Fig. 2. Effect of NR on cytosolic chaperones and markers of ER stress in primary mice chondrocytes.

(**a**) mRNA levels of cytosolic chaperones following treatment with NR. NS=not significant compared with the control group; n=3. (**b**) ER stress proteins levels (i.e. calreticulin, Grp78 and Grp94) after treatment with NR.

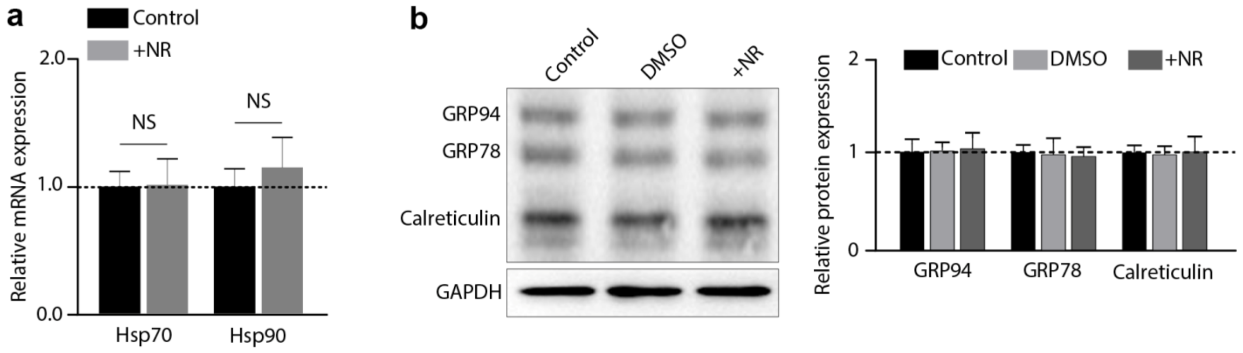

Supplementary Fig. 3. Effect of silencing Atf5 in primary mice chondrocytes.
(a) Effect of silencing Atf5 on the mRNA levels of UPR^{mt} markers in primary mice chondrocytes following treatment with IL-1β and nicotinamide riboside (NR). *p<0.05; n=3. **(b)** The rate of TUNEL positive cells in different groups. *p<0.05, NS=not significant; n=3. **(c)** The cell proliferation rate in different groups. *p<0.05, NS=not significant; n=3. **(d-e)** The relative fluorescence intensity of MMP13 **(d)** and Col2A1 **(e)** in different groups. *p<0.05, NS=not significant; n=3.

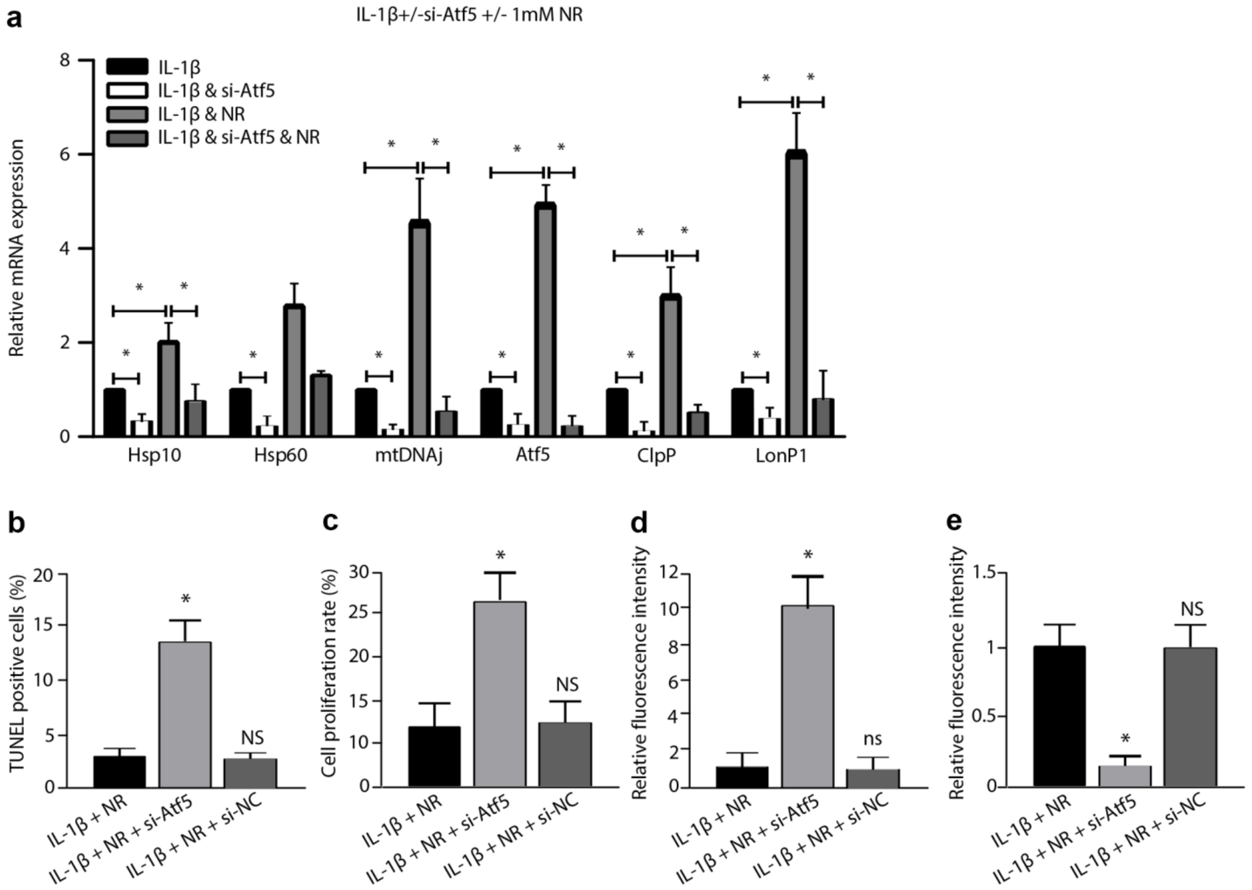

Supplementary Fig. 4. Effect of nicotinamide riboside (NR) on UPR^{mt}, cytosolic stress responses and ER stress.

Effect of NR or DMSO on UPR^{mt} markers (**a**), cytosolic chaperones (**b**) and ER stress proteins levels (i.e. calreticulin, Grp78 and Grp94) (**c**) in mice subjected to DMM surgery or a sham procedure. *p<0.05 compared with the sham group; #p<0.05 compared with the DMM group; NS=not significant. n=6-8 per group. (**d-g**) Changes in spontaneous activity, including paw withdrawal response thresholds (**d**); distance of locomotion, meters in 12h (**e**); speed of locomotion (**f**) and rearing frequency, times in 12h (**g**); were evaluated using the Laboratory Animal Behavior Observation Registration and Analysis System (LABORAS) 8 weeks after DMM surgery. *p<0.05, NS=not significant, n=6-8.

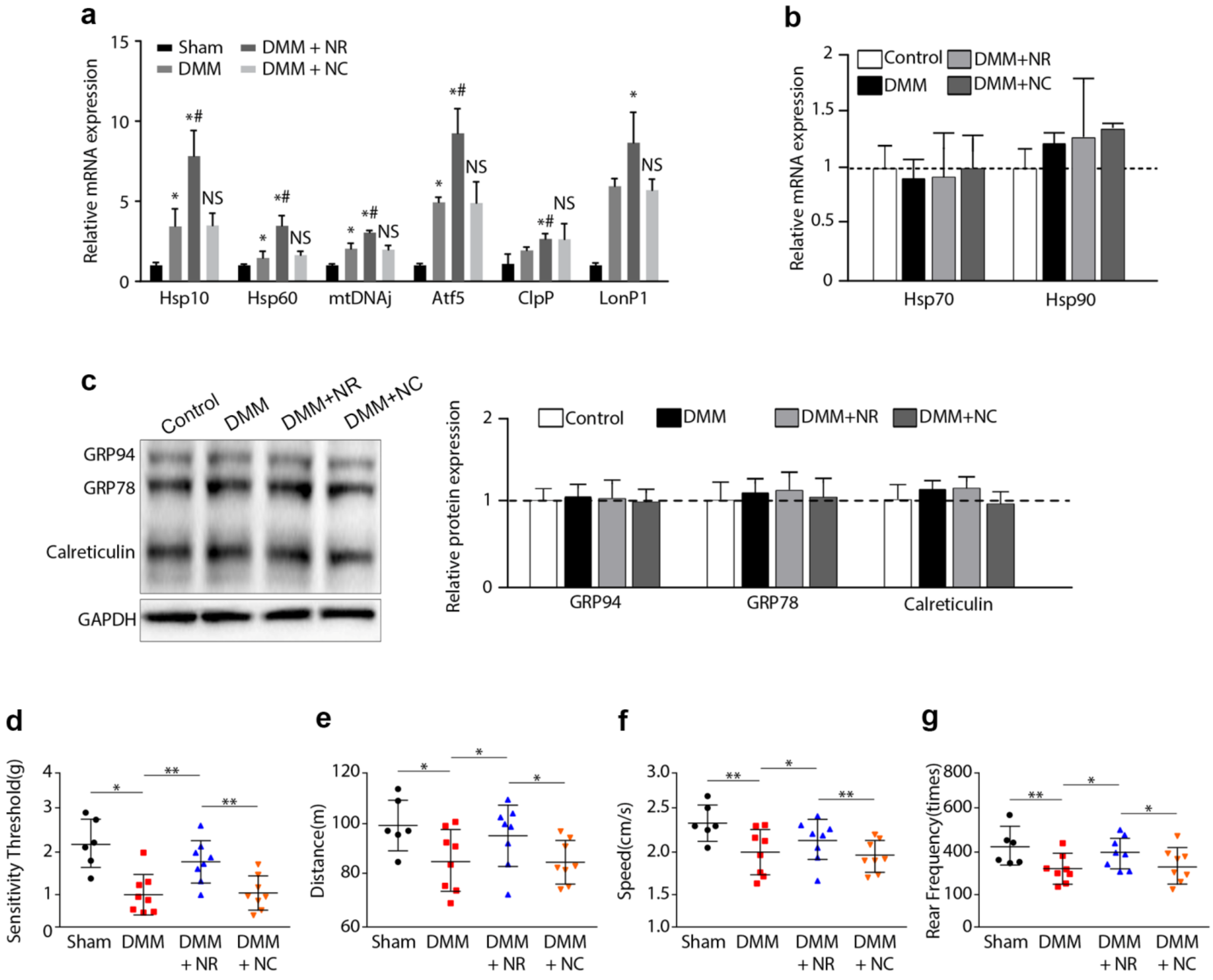

Supplementary Fig. 5. Conditional knockout of Atf5 suppressed the activation of UPR^{mt} in mice.

(**a**) Effect of conditional knockout of Atf5 on UPR^{mt} markers. *p<0.05; n=6. (**b**) Effect of NR treatment on UPR^{mt} markers in *ATF5$^{f/f}$Col2a1-CreER^{T2}* mice and *ATF5$^{f/f}$* mice subjected to DMM surgery. compared with the sham group; *p<0.05, NS=not significant. n=6.

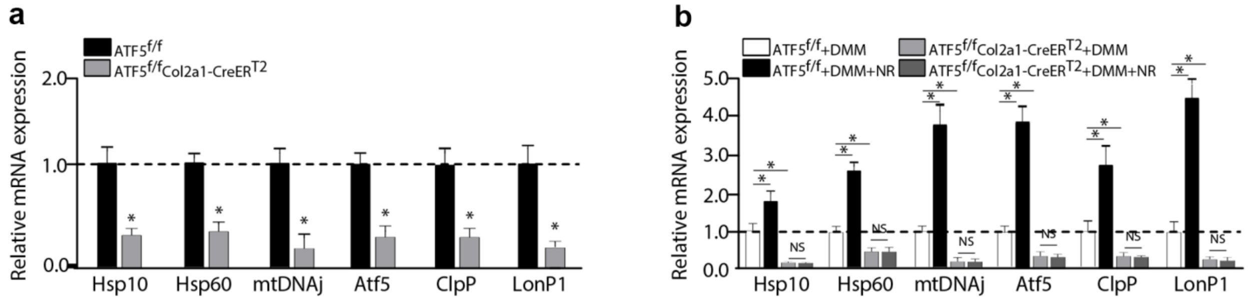

Supplementary Fig. 6. Clinical characteristics of OA patients with lower or higher UPR^{mt} markers.

(**a**) Representative pictures of H&E-stained cartilage. Scale bar, 500 µm. (**b-c**) qPCR and Western blot analysis of MMP13, Col2a1, ADAMTS5 and Aggrecan expression in cartilage tissues from patients in different groups. ***p< 0.001; n=20. (**d-f**) Comparison of WOMAC score of function and stiffness and ICOAP score between OA patients with lower or higher UPR^{mt} markers. WOMAC score of function (**d**); WOMAC score of stiffness (**e**); Constant and intermittent pain score of ICOAP (**f**); *p<0.05, n=9-11.

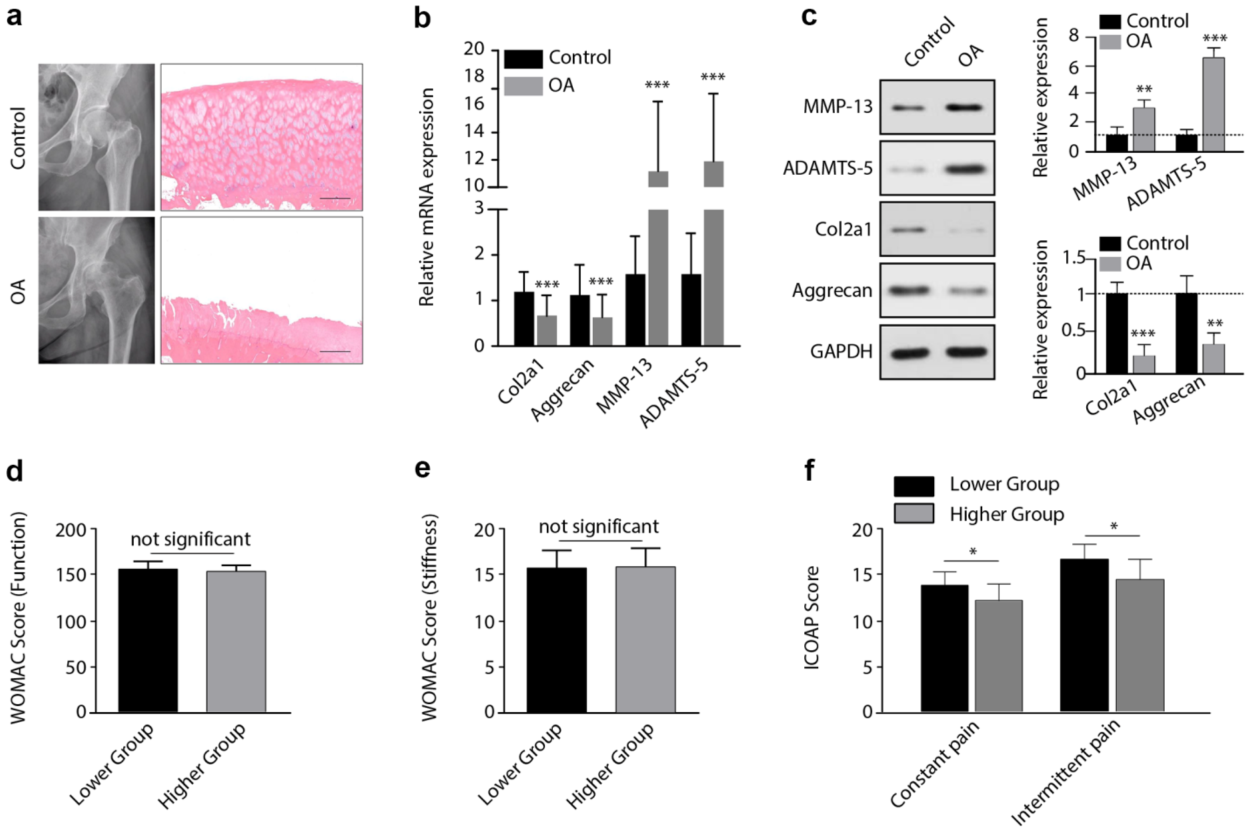

Supplementary Table 1. Primer sequences for RT-PCR.

Gene	Forward (5'-3')	Reverse (5'-3')
**Hsp10 (mouse)**	GGCCCGAGTTCAGAGTCC	TGTCAAAGAGCGGAAGAAACTT
**Hsp60 (mouse)**	CAGAGCTGGGTCCCTCACT	CTGTGGGTAGTCGAAGCATTT
**ClpP (mouse)**	GGTTGAGAATGTAGCCCATGA	CGATGATATCCCGAATGGTC
**LonP1(mouse)**	GGTTGAGAATGTAGCCCATGA	CGATGATATCCCGAATGGTC
**mtDNAj (mouse)**	AGTCACCCACACAAGCACTG	CCAGCCTCTCGCCTATCC
**Atf5 (mouse)**	TCCGCTCACACCGTCTCT	AAGGCGAAGGTGGAGGAC
**Hsp70 (mouse)**	CTACAAGGCGGACGA	TAGGACTCGAGCGCATTCTT
**Hsp90 (mouse)**	TTTCGTGCGTGCTCATTCT	AAGGCAAAGGTTTCGACCTC
**Hsp10 (human)**	AGGTGGCATTATGCTTCCAG	TGACAGGCTGAATCTCTCCAC
**Hsp60 (human)**	GATGGAGAAGCTCTAAGTACACTCG	GCTGGTTCTTTCTATTGTCACCA
**ClpP (human)**	CCCGTATCATGATCCACCA	AGAGCTGCTTCTTGAGCTTCAT
**LonP1(human)**	GGTTGAGAATGTAGCCCATGA	TCACGATCTCTGCAGTCAGG
**mtDNAj (human)**	GAGGACGAGACAGATGTGGAG	TCCTGCGGAGCTATCCAT
**Atf5 (human)**	TTTGCAGTGCGGGAAGAT	AAAATGAACACCCAGTCACCA

Supplementary Table 2. Clinical characteristics of patients in this study.

Characteristic		Patients with femoral neck fracture (n=20)	OA patients with higher UPR^{mt} levels (N=11)	OA patients with lower UPR^{mt} levels (N=9)
Age, years, mean±SD		69±5.9	67±9.7	65±6.9
Gender (male/female)		11/9	7/4	6/3
BMI, kg/m^2, mean±SD		28.7±3.17	28.09±3.6	27.2±2.53
Kellgren & Lawrence grade IV		0	11	9
Duration of hip OA symptom (month)		0	7.6±5.9	7±4.8
*Frequent pain medical consumption, n(%)	NSAID	0	6 (54.5%)	5 (55.6%)
	Opiates	0	2 (18.2%)	2 (22.2%)

OA, osteoarthritis; UPR^{mt}, mitochondrial unfolded protein response; BMI, body mass index; NSAID; Non-Steroidal Anti-Inflammatory Drugs; *Frequent pain medical consumption, 3–5 times/week or daily use in the past 3 weeks.
